# Direct Conversion of the Biomass-Derived Acetone–Ethanol Mixture into Propene over Zr/Beta Zeolite

**DOI:** 10.1021/cbe.5c00025

**Published:** 2025-06-26

**Authors:** Mengting Zhang, Ruxin Li, Jun Yu, Weili Dai

**Affiliations:** School of Materials Science and Engineering, 12538 Nankai University, Tianjin 300350, China

**Keywords:** MPV reduction, Zr/Beta, propene, acetone-ethanol conversion, Lewis acid sites

## Abstract

The conversion of biomass fermentation liquor has garnered significant attention due to its potential for sustainable chemical production. Particularly, the transformation of an acetone–ethanol mixture, derived from the separation of high-value butanol, into other valuable compounds represents a critical advancement in biorefinery processes. Herein, we present a high-efficiency Zr/Beta zeolite catalyst for the conversion of an acetone-ethanol mixture into propene. Through systematic optimization, the optimal catalyst 5%Zr/Beta achieves a high propene yield (37.8%) with a propene selectivity of 67%. Spectroscopic results reveal that the conversion of acetone and ethanol primarily proceeds via the Meerwein–Ponndorf–Verley (MPV) reduction at Zr sites to form the isopropanol intermediate, followed by acid-catalyzed dehydration to propene facilitated by Si–OH groups. The high propene selectivity is due to the minor side reaction of converting acetone to isobutene, accompanied by the accumulation of cyclic unsaturated aldehydes/ketones and aromatic compounds deposited on the Zr active sites, leading to catalyst deactivation. Additionally, the Zr/Beta catalyst demonstrates good regenerability, which could recover to the initial state after a facile calcination process in air. This work offers a promising approach for the synthesis of propene from a biomass-derived acetone-ethanol mixture, contributing to the development of sustainable catalytic processes for biorefinery applications.

## Graphical abstract

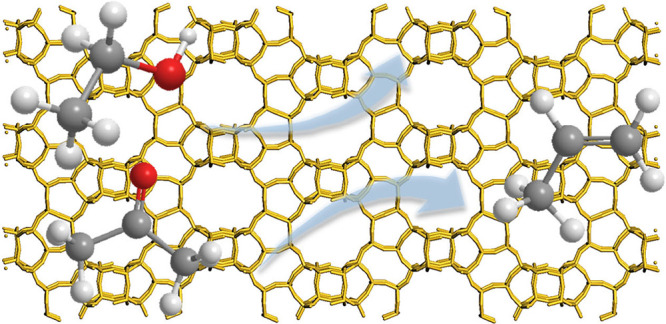

## 1 Introduction

The escalating global energy crisis and environmental concerns have driven intensive research efforts toward sustainable and renewable energy alternatives. Among these, biomass-derived energy has been perceived as a promising candidate owing to its inherent renewability and minimal environmental impact. Therefore, the catalytic transformation of biomass into advanced biofuels and value-added platform chemicals has evolved into a critical area of research in both chemistry and engineering. One particularly notable process is the acetone-butanol-ethanol (ABE) fermentation, derived from starchy feedstocks,^1^ which offers a viable pathway for producing compatible fuels. This has led to a surge in research focused on ABE fermentation and the upgrading of its components into ready-to-use fuels and high-value chemicals. Anbarasan et al.^2^ proposed a novel concept of integrating biofermentation and chemical catalysis, converting ABE mixture into C_5_–C_15_ hydrocarbons. A critical aspect of this process is maintaining the suitable carbon chain length of intermediates for aviation fuels.^3–5^ However, the reliance on noble metal catalysts for oxidation/reduction reactions^6–8^ and bases for alcohol condensation^2,9^ poses challenges, including equipment corrosion, wastewater contamination, and catalyst recovery issues.

To address these limitations, bifunctional catalysts such as Zn-HZSM-5 and Ga-HZSM-5 have been developed, achieving high aromatics yields (up to 77%) from the ABE mixture.^10,11^ Similarly, Canizares et al. developed HZSM-5 zeolites with a varied SiO_2_/Al_2_O_3_ ratio for the conversion of acetone and 1-butanol into C_1_–C_10_ hydrocarbons, with increasing aromatic and gaseous paraffin production at high temperatures.^12^ Despite the research endeavor devoted, the separation of 1-butanol from the ABE mixture, driven by its rising demand and price^13^ often leads to increased industrial costs due to the subsequent separation of acetone and ethanol. While extensive research has been conducted on the individual catalytic conversion of acetone or ethanol, the direct utilization of the acetone-ethanol mixture derived from ABE fermentation remains underexplored. Meerwein–Ponndorf–Verley (MPV) reduction has garnered attention in this field with high selectivity for C=O functional groups in unsaturated carbonyl compounds using secondary alcohols as hydrogen donors.^14^ Zirconium-based catalysts have shown promise with the advantages of moderate acidity and oxidizing capabilities. The modification of ZrO_2_ through impregnation with active phases has been found to change surface properties and enhance intrinsic activity/selectivity in MPV reactions.^15–17^ Moreover, it has been reported that ethanol has been successfully converted to propene via MPV reactions over AgCeO_2_ and ZrO_2_/SiO_2_ catalysts, in which acid properties play a crucial role in the reaction process.^18^

Traditional propene production via steam cracking, catalytic cracking, propane dehydrogenation, and methanol-to-olefins processes dominates the petrochemical industries; their high carbon emission and reliance on fossil feedstocks necessitate sustainable alternatives. However, the production of propene from biomass fermentation has remained largely unexplored. Herein, we present a breakthrough in biorefinery catalysis through Zr/Beta zeolite that converts a biomass-derived acetone-ethanol mixture to propene via tandem MPV reduction-dehydration. The effects of the support material, Zr loading, reaction temperature, and feed composition on the catalytic performance were systematically investigated. Under optimized conditions, a propene selectivity of 67% is achieved over a 5% Zr/Beta catalyst. Furthermore, the reaction network and catalyst deactivation pathways were elucidated through online mass spectrometry and spectroscopy analysis. These findings provide valuable insights into the sustainable production of propene from biomass-derived feedstocks.

## 2 Experimental Section

### 2.1 Catalyst Preparation

Commercial H-Beta zeolite with a nominal nSiO_2_/nAl_2_O_3_ ratio of 25 was utilized as a precursor in the production of Si-Beta catalysts. To obtain dealuminated Si-Beta, H-Beta zeolite was added to a nitric acid solution with continuous stirring at 373 K for 12 h. The above product was separated by filtration and washed with deionized water until the solution was neutral. Afterward, the resulting powder was dried at 473 K overnight. Completely dealuminated Si-Beta was used to synthesize Zr/Beta through the impregnation method by employing zirconyl nitrate. Typically, the metal precursor was dissolved in an appropriate amount of water, and 1.0 g of Si-Beta was then added, and the mixture was stirred for 8 h. The slurry was fully mixed and dried by evaporation in a hot water bath at 353 K. The dried sample was calcined in air at 823 K for 6 h. ZrO_2_ used as the reference sample was purchased from Aladdin.

All other metal catalysts (Sc, Y, La, Ce, and Zn) loaded on the Si-Beta were prepared using the same method described above, with the corresponding metal nitrates as precursors. Other Zr catalysts with aluminum-free substrates (S-1, MCM-41, SiO_2_) were also synthesized following the same procedure.

### 2.2 Characterization Techniques

The powder X-ray diffraction (XRD) patterns of samples were recorded on a Bruker D8 diffractometer with Cu Kα radiation (λ = 0.1541 nm) at a scan rate of 4° min^–1^ in the region of 2θ = 5–50°.

Mapping images of the elemental distribution of zeolite crystals was conducted under the high-angle annular dark field scanning transmission electron microscopy (HAADF-STEM) mode using FEI built-in energy-dispersive spectrum (EDS) software.

Diffuse reflectance infrared frontier (DRIFTS) spectra of the catalysts were measured on a Bruker Tensor 27 spectrometer with 128 scans at a resolution of 2 cm^–1^. A self-supporting pellet made of the catalyst was placed in the reaction chamber and pretreated in flowing dry air at 673 K for 1 h. The DRIFTS spectra were recorded in dry air with KBr as the background.

The diffuse-reflectance ultraviolet–visible (UV–vis) spectra of the Zr/Beta zeolites were recorded on a PerkinElmer Lambda 750 UV–vis spectrophotometer (200–800 nm) using BaSO_4_ as a background.

X-ray photoelectron spectroscopy (XPS) measurements were conducted on an ESCALAB 250Xi spectrometer manufactured by Thermo Fisher Scientific to obtain the valence states. When the vacuum degree is higher than 10^–7^ Pa, Al–K_α_ (hν = 1486.6 eV) is used as the X-ray source with an operating voltage of 13 kV and a power of 250 W. The photoelectron peak of C 1*s* (binding energy is 284.6 eV) is used as the reference energy, and the error is ± 0.1 eV.

The carbon deposits in the catalysts were analyzed by the TAQ600 thermogravimetric analyzer (TGA). The profile was recorded in a 20% O_2_/N_2_ flow from 298 to 1073 K with a heating rate of 10 K min^–1^.

### 2.3 Catalytic Evaluations of Zeolite Catalysts

The conversion of the acetone–ethanol mixture was performed in a fixed-bed reactor at atmospheric pressure. Typically, 0.15 g of catalyst with the sieve fraction particles of 0.25–0.5 mm was placed in a quartz reactor (5 mm i.d.) and activated in a nitrogen atmosphere (20 mL min^–1^) at 723 K for 1 h. After cooling to the reaction temperature, the AE flow (the volume ratio of acetone to ethanol is 3:1) was introduced into the reactor by a *K*_d_ Scientific syringe pump to obtain a weight hourly space velocity (WHSV) of 1 h^–1^. The hydrocarbon products were separated by the plot-q column (30 m × 0.32 mm × 20 μm) and then analyzed by online gas chromatographs equipped with flame ionization detector (FID) detector. The conversion of the acetone–ethanol mixture and product selectivity were calculated according to eqs 1 and 2.

$$\mathrm{Conversion}\;(\mathrm{carbon}\;\mathrm{mol}\%)=\frac{[\mathrm{AE}{]}_{\mathrm{inlet}}-[\mathrm{AE}{]}_{\mathrm{outlet}}}{[\mathrm{AE}{]}_{\mathrm{inlet}}}\times 100\%$$ 1

$$\mathrm{Product}\;\mathrm{selectivity}\;(\mathrm{carbon}\;\mathrm{mol}\%)=\frac{C\;\mathrm{atoms}\;\mathrm{in}\;\mathrm{product}*[\mathrm{product}{]}_{\mathrm{outlet}}}{\sum C\;\mathrm{atoms}*[\mathrm{product}{]}_{\mathrm{outlet}}}\times 100\%$$ 2

During the investigation of different catalysts, WHSV and monocomponent experiments, the catalytic performance processes were as described above, with only one factor changed, unless the conditions were specifically specified.

During the catalyst regeneration process, the catalyst was calcined in air at 823 K for 6 h and used for the next reaction.

### 2.4 Acetone–Ethanol Online MS Spectra Experiments

Acetone–ethanol online MS spectra experiments were performed in the same fixed-bed reactor connected with a downstream gas sampling mass spectrometer (MS, Pfeiffer-Balzer Omnistar). Typically, helium is utilized as the carrier gas since it has a low *m*/*z* value of 4, which has no overlap with the main products or intermediates in acetone–ethanol conversion. For experiments, 0.15 g of samples were pretreated at 723 K for 1 h in a helium flow (20 mL min^–1^) and then cooled to 298 K. Subsequently, the acetone–ethanol mixture was continuously introduced to the catalyst through a bubbler at 623 K. The gas-phase products were monitored by online MS, referred to the database of the National Institute of Standards and Technology (NIST). In terms of *m*/*z* values, the principle of choice is evading contributions from other products but still obtaining a high intensity. If not avoided, the mass peak could be corrected for the mass intensity.

## 3 Results and Discussion

### 3.1 Catalyst Characterization

The Zr/Beta catalysts were synthesized via wetness impregnation, followed by a procedure described in detail in our previous work.^19,20^ The structural evolution of Beta before and after Zr incorporation was characterized using XRD, DRIFTS, UV–vis, and TEM (Figure 1). The XRD patterns (Figure 1a) show the retention of the BEA topology in both parent Si-Beta and Zr-modified samples, indicating the preservation of the zeolite framework during acidic post-treatment. The absence of distinct ZrO_2_ peaks (ZrO_2_, PDF#37-1484) in Zr/Beta catalysts with low Zr loadings (≤10%) implies the uniform dispersion of Zr species within the matrix. According to our previous work, the dealumination of H-Beta and the incorporation of metal heteroatoms are closely associated with changes in silanol groups during post-synthesis treatment.^19,20^ As illustrated in Figure 1b, the untreated H-Beta exhibits two main spectral bands at 3740 and 3600 cm^–1^, corresponding to the isolated Si–OH groups and the bridging Si–OH–Al groups, respectively.^21,22^ After nitric acid treatment, the removal of framework Al results in the disappearance of these Al-related bands of 3600 cm^–1^. In addition, the band intensity of the 3740 and 3520 cm^–1^ increases, corresponding to isolated internal Si–OH groups and hydrogen-bonded silanol groups at the framework defects, respectively.^23,24^ The introduction of Zr species leads to a decrease in the band intensity of these Si–OH groups (Figure 1b), suggesting an interaction between Zr and the silanol groups and the successful incorporation of Zr into the Beta zeolite framework. UV–vis spectroscopy was further employed to investigate the coordination environment of the doped Zr species. As shown in Figure 1c, the UV–vis spectra of the Zr/Beta samples exhibit an absorption band centered at 205 nm, which is attributed to the ligand-to-metal charge transfer from O^2–^ to tetrahedrally coordinated Zr^4+^ ions.^25–27^ This indicates that the majority of Zr species are incorporated into the Beta zeolite framework in a tetrahedral coordination environment, which is expected to serve as catalytically active Lewis acid sites.^26^ The TEM image and elemental mapping of the Zr/Beta sample (Figure 1d) reveal that the Zr species are homogeneously distributed in the catalyst without significant aggregation of Zr.

**Figure 1:**
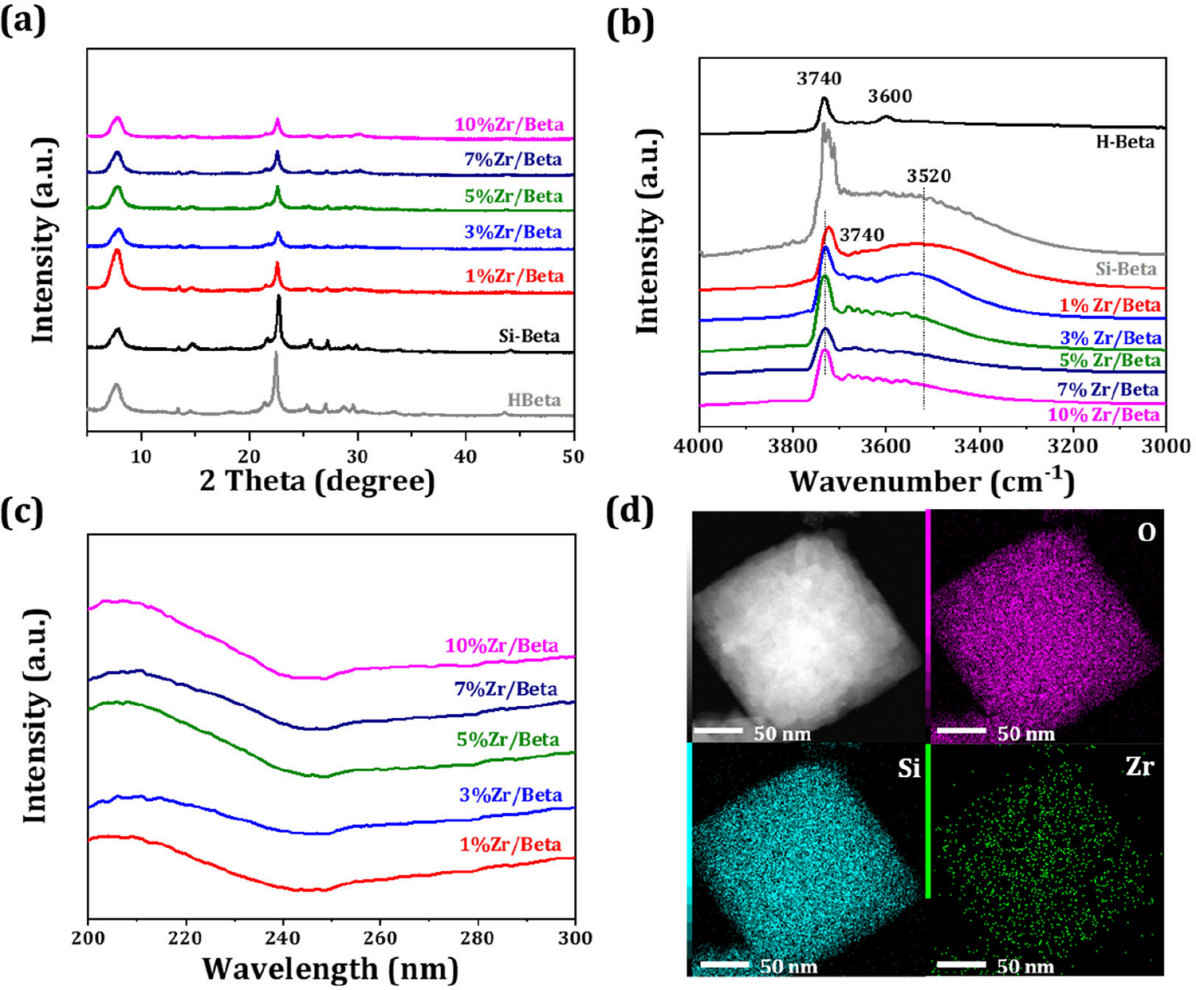
XRD patterns (a), DRIFT spectra (b), and UV–vis spectra (c) of H-Beta, Si-Beta and Zr/Beta catalysts; TEM images of 5%Zr/Beta with corresponding element mapping (d).

### 3.2 Catalytic Performance of Zeolite Catalysts in Acetone–Ethanol Mixture Conversion

To evaluate the catalytic performance of various Lewis acid catalysts in the conversion of an acetone-ethanol (AE) mixture, we prepared a series of samples with frequently used metals as active centers and Si-Beta zeolites as supports. As summarized in Table 1, the introduction of Lewis acid sites significantly enhances the reaction efficiency in comparison with Si-Beta zeolite, and all metal-based zeolite catalysts exhibit notable catalytic activity. Among these catalysts, Zr-based zeolite emerges as the most effective candidate for the MPV reaction, consistent with previous reports.^28,29^ The Zr/Beta catalyst demonstrates superior performance in the acetone/ethanol mixture conversion, achieving the highest propene yield (37.8%). This result underscores the exceptional activity of Zr in facilitating the MPV reaction and highlights the potential of Zr/Beta as a highly efficient catalyst for this transformation.

**Table 1:** Acetone–Ethanol Mixture Conversion over Various Catalysts^a^

				product selectivity (mol %)				
catalyst	*T* (K)	conversion (%)	C_3_H_6_ yield (%)	C_2_H_4_	C_3_H_6_	CH_3_CHO	C_4_H_6_	others
H-Beta	623	80.9	11.6	34.5	14.3	0	42.8	8.4
semi del/Beta	623	57.0	6.6	49.5	11.6	0.4	36.0	2.5
Si-Beta	623	25.7	3.1	76.5	12.0	5.1	1.7	4.6
5%Sc/Beta	623	50.4	34.1	7.6	67.7	2.5	10.1	7.1
5%Y/Beta	623	46.6	31.3	11.2	67.2	6.4	6.7	8.5
5%La/Beta	623	36.7	23.2	5.2	63.2	10.2	3.9	17.6
5%Ce/Beta	623	39.4	28.7	9.7	72.8	6.5	4.1	6.9
5%Zn/Beta	623	34.1	17.7	26.9	51.8	14.4	4.0	2.9
5%Zr/Beta	623	53.6	37.8	2.5	67.2	5.3	5.4	19.6

a Reaction conditions: acetone: ethanol = 3:1, WHSV = 1.0 h^–1^, TOS = 1 h.

Subsequently, the catalytic performance of Zr/Beta, Si-Beta, and ZrO_2_ in the conversion of an acetone–ethanol mixture to propene was evaluated in a fixed-bed reactor at 623 K for 6 h. As illustrated in Figure 2a,b, the mixture conversion of these three catalysts almost unchanges with prolonged time, while the product distribution diverges markedly:, the product distribution diverges markedly: the primary product of the Zr/Beta-catalyzed reaction is propene (Figure 2b), whereas Si-Beta favors ethanol dehydration to produce ethylene (76.5% selectivity), highlighting the role of silanol groups (Si–OH) dominating in this process. In contrast, the formation of ethylene, acetaldehyde, and other oxidized formyl compounds over the ZrO_2_ sample suggests that acetone undergoes aldol condensation on ZrO_2_ without further cleavage of the formyl groups.^30^ These results highlight the critical role of silanol groups in the post-MPV reaction steps. To further investigate the influence of zeolite pore architecture on catalytic performance, four aluminum-free materials with distinct structural characteristics (S-1, MCM-41, SiO_2_, and Si-Beta) were functionalized with Zr and tested in acetone-ethanol conversion. Figure 3a shows a similar catalytic performance over these Zr-modified catalysts, suggesting a limited correlation between the three-dimensional pore topology and reaction outcomes. The propene production pathway predominantly involves the MPV reduction of acetone followed by a dehydration process, reaffirming the synergistic effect between Si–OH groups and Zr in facilitating propene formation.^18^

**Figure 2:**
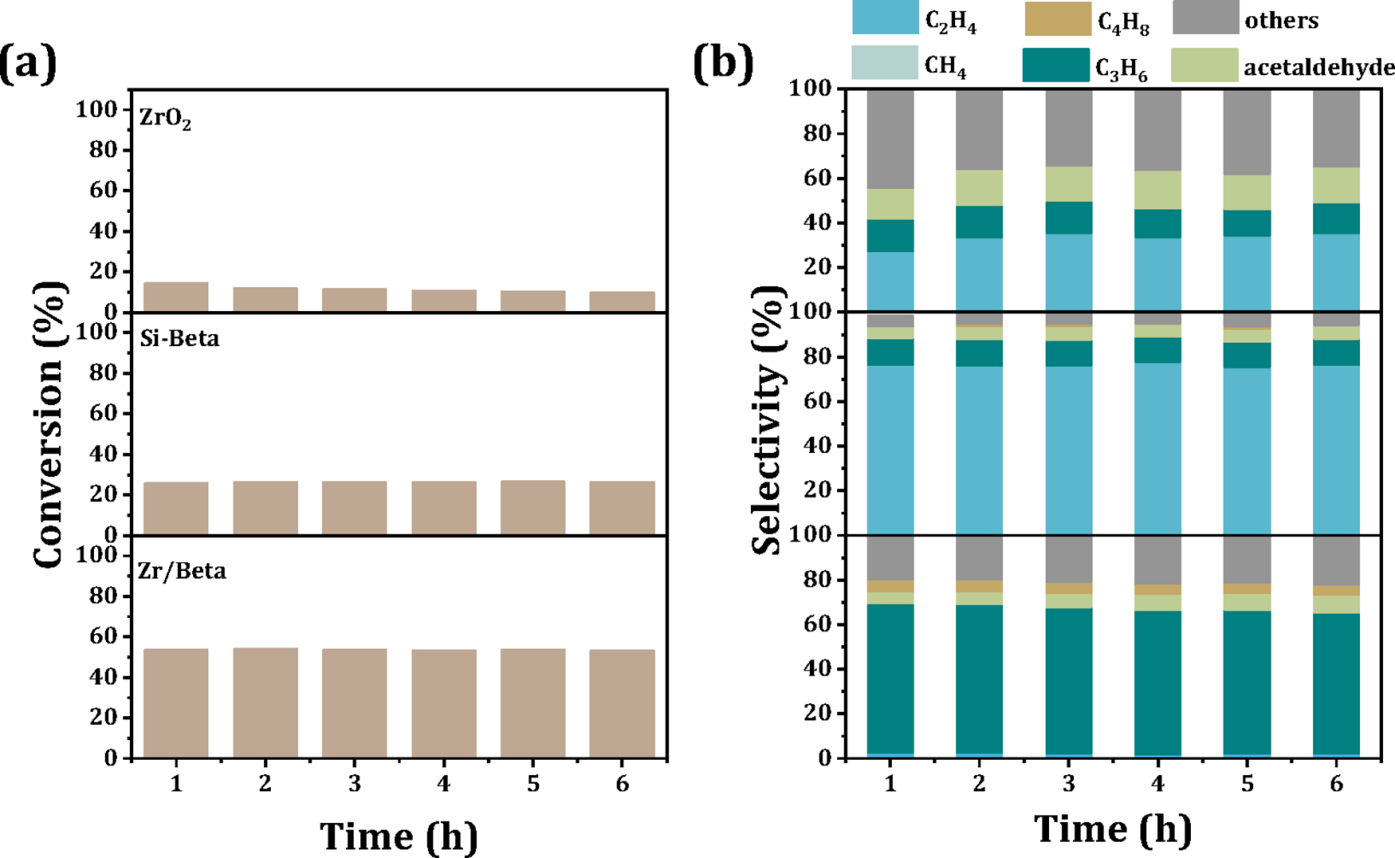
Acetone-ethanol mixture conversion (a) and product selectivity (b) in the acetone-ethanol mixture conversion over Zr/Beta, Si-Beta and ZrO_2_ catalysts. Reaction conditions: acetone: ethanol = 3:1, reaction temperature = 623 K, WHSV = 1.0 h^–1^, and TOS = 6 h.

**Figure 3:**
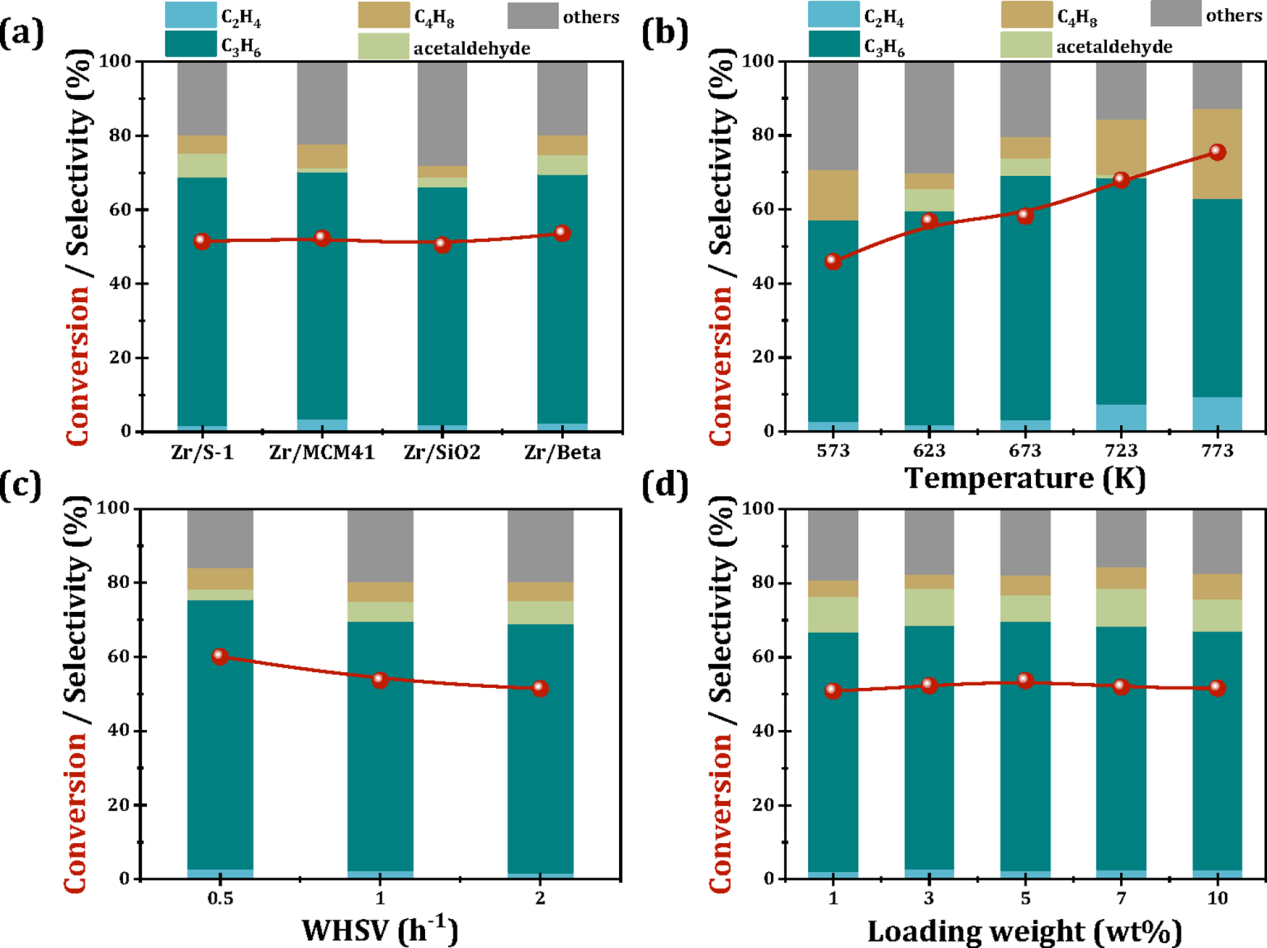
Acetone–ethanol mixture conversion and product selectivity over different zeolites (a), Zr/Beta catalyst with different temperature (b), WHSV (c), and Zr loading (d). Reaction conditions: acetone: ethanol = 3:1, TOS = 1 h.

The methodical examination of reaction parameters, including operating temperature, WHSV, and Zr loading on the catalytic behavior of Zr-Beta catalysts, was systematically investigated. As depicted in Figure 3b, the conversion of the acetone–ethanol mixture demonstrates monotonic enhancement from 573 to 823 K. The results indicate that higher reaction temperature accelerates the MPV reaction and subsequent dehydration process. However, the propene yield declines at elevated temperatures, accompanied by an increase in C4 olefin production (Figure 3b), which indicates that acetone undergoes aldol condensation to form C4 compounds over Zr/Beta, in line with previous studies.^30,31^ The influence of WHSV on the catalytic performance of Zr-Beta is shown in Figure 3c, the mixture conversion and propene selectivity decrease slightly as the WHSV increases from 0.5 to 1 h^–1^ but remain stable at a WHSV value up to 2 h^–1^. This suggests that within a specific WHSV range, the Zr active sites on Zr/Beta effectively facilitate the MPV reaction and dehydration to produce propene. Similarly, by changing the Zr loading (Figure 3d), mixture conversion and propene selectivity display no significant change, indicating that even a low Zr loading of 1% is sufficient to promote the MPV reaction and subsequent dehydration with the assistance of framework Si–OH groups.

### 3.3 Catalytic Modulation Experiments with the Monocomponent of the Acetone–Ethanol Mixture

Given the complexity of the catalytic performance of the acetone–ethanol mixture at high temperatures and the difficulty in identifying the reaction pathways,^10^ this study aims to elucidate the role of Zr in the conversion of the acetone–ethanol mixture and the underlying reaction mechanism. Detailed investigations of the substrate conversion and product selectivity of individual components over the 5% Zr/Beta catalyst were conducted. As illustrated in Figure 4a,b, ethanol achieves complete conversion (>99%) in all tested temperatures (573–773 K), predominantly yielding ethylene via ethanol dehydration. In contrast, acetone exhibits low conversion (<25%) below 673 K, with high isobutylene selectivity. The transformation pathway for isobutylene formation from acetone proceeds through three concerted steps: (i) condensation of dual acetone molecules into diacetone alcohol via the enol intermediate, (ii) dehydration of diacetone alcohol to form phorone, and (iii) cleavage of phorone at external framework Lewis acidic Al sites to generate isobutylene.^31,32^ Therefore, the production of C4 olefins is observed during the conversion of acetone. As the temperature increases, the acetone conversion rises progressively, while the C4 olefin selectivity declines slightly due to the isomerization and cracking of isobutylene, which is consistent with our previous study.^30^ Notably, different catalytic behavior is observed in the acetone-ethanol co-feed system. In this case, both substrate conversion and propene selectivity exhibit positive temperature dependence up to 723 K. However, at 773 K, a slight decrease in propene selectivity occurs, which is attributed to the additional conversion of excess acetone into C4 olefins, aligning with the results obtained from the acetone-only conversion experiment. As depicted in Figure 4c, the acetone-ethanol conversion and propene selectivity remain almost stable throughout the AE mixture conversion process. In contrast, for the monocomponent reaction (ethanol or acetone), both substrate conversion and main product selectivity decay sharply along with time, implying the progressive coverage of the active sites on the 5% Zr/Beta catalyst, which ultimately leads to catalyst deactivation.

**Figure 4:**
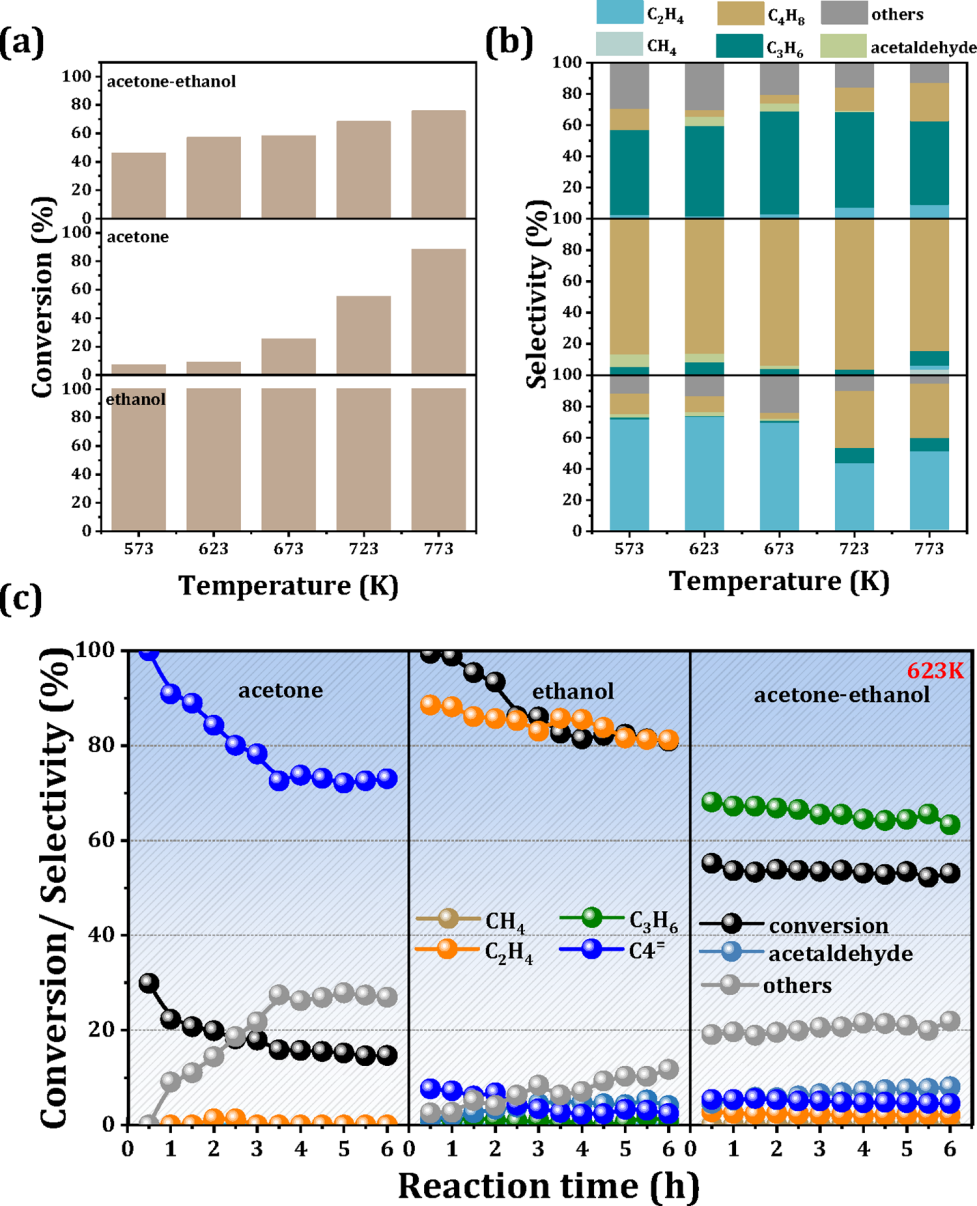
Monocomponent and mixture of acetone-ethanol conversion (a) and product selectivity (b) over 5%Zr/Beta catalyst. Reaction conditions: 573–773 K, WHSV = 1.0 h^–1^. (c) Time-dependent monocomponent and acetone-ethanol mixture conversion and product selectivity over 5%Zr/Beta catalysts. Reaction conditions: acetone: ethanol = 3:1, WHSV = 1.0 h^–1^, 623 K, and TOS = 6 h.

Furthermore, we systematically investigated the effects of acetone-ethanol mixture composition on conversion and product selectivity, as presented in Figure 5. It demonstrated that the acetone/ethanol ratio significantly influences both conversion efficiency and product distribution. Under ethanol-rich conditions (acetone: ethanol <1:1), propene and C4 olefins constitute the predominant products. Conversely, acetone-dominant mixtures (acetone: ethanol >1:1) suppress overall conversion while enhance propene selectivity. Strikingly, as acetone-to-ethanol ratio is 1:1, nearly complete conversion (∼100%) is achieved, with ∼50% propene selectivity. Minor quantities of C4 olefins and other oxygenated compounds are also observed, which likely originates from the partial conversion of acetone to acetic acid over Lewis acid sites.^31,33^

**Figure 5:**
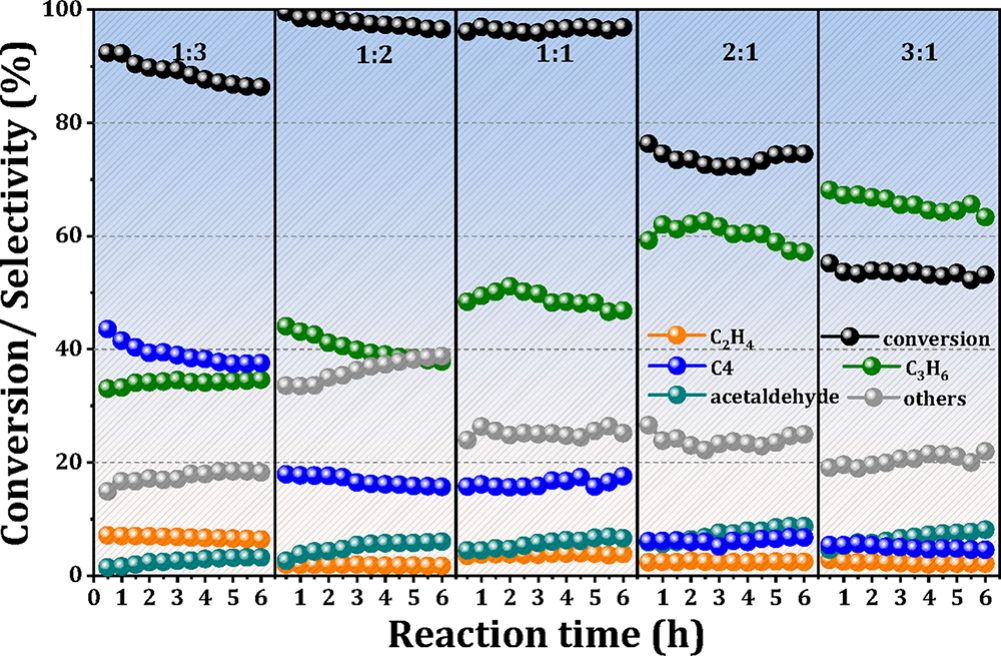
Catalytic performance of AE mixture conversion with different acetone-to-ethanol ratios over the 5%Zr/Beta catalyst. Reaction conditions: temperature = 623 K, WHSV = 1.0 h^–1^, and TOS = 6 h.

To further evaluate the recyclability of the 5%Zr/Beta catalyst, the long-term stability experiment was performed. As shown in Figure 6, the propene selectivity retains above 60% during the initial 80-h operational period. Nevertheless, catalyst deactivation is observed with prolonged time-on-stream due to the formation of acetals, the cyclization of excess acetone, and the accumulation of oxygenated compounds, aromatics and their derivatives,^34^ which is verified by thermogravimetric analysis (TGA) of the spent catalyst (Figure 7a). The TGA profile shows two distinct weight loss regions: the low-temperature region (<500 K) is associated with water desorption, and the high-temperature region (>500 K) corresponds to the removal of organic deposits. Quantitative analysis shows that the coke content increases from 5.7 wt % (0.5 h) to 9.5 wt % (110 h), indicating the gradual accumulation of organic species on the catalyst surface and leading to catalyst deactivation. After a facile calcination treatment in air for 6 h, the catalytic performance recovers to the initial state with ∼49.2% conversion and ∼63.3% propene selectivity (Figure 6). XRD and XPS characterization studies of the fresh, used, and regenerated catalysts reveal no significant structural change and the preservation of the Zr oxidation state (Figure 7b,c). The characteristic peaks of Zr 3*d*_5/2_ (182.9 eV) and Zr 3*d*_3/2_ (185.3 eV) remain unchanged, and the XPS spectra confirm the stability of Zr species throughout the reaction and regeneration processes.^20,35^ Additionally, no significant aggregate of Zr species is observed on the regenerated catalyst compared to the fresh one (Figures 7d and 1d), further demonstrating the stability of Zr species. Based on these findings, we speculate that catalyst deactivation is primarily caused by carbon deposition, which gradually covers active sites or blocks pores.

**Figure 6:**
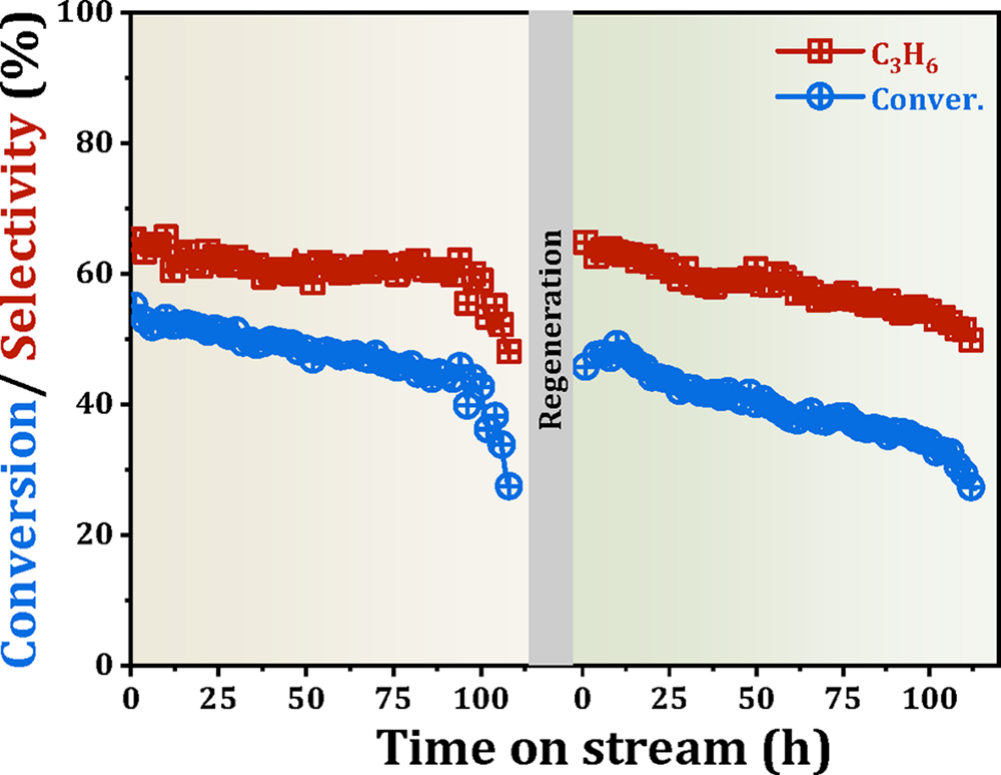
Stability tests of the 5%Zr/Beta catalyst in the AE-to-propene conversion. Reaction conditions: acetone: ethanol = 3:1, WHSV = 1.0 h^–1^, 623 K.

**Figure 7:**
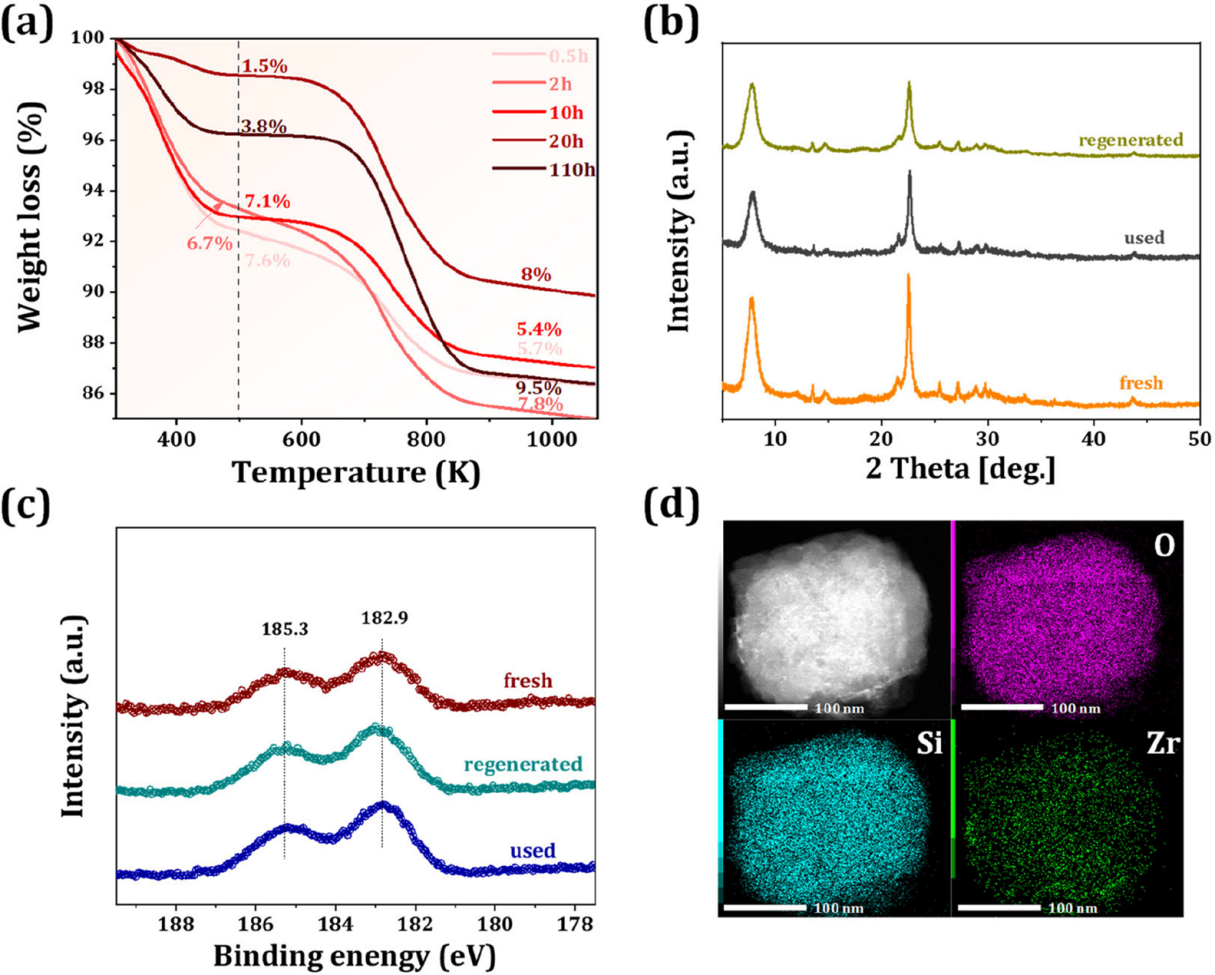
(a) TGA curves of the used 5%Zr/Beta catalysts after the reaction with different TOS of 0.5 ∼ 110 h, WHSV = 1.0 h^–1^; (b) XRD patterns, (c) The Zr 3*d* XPS of fresh, used and regenerated 5%Zr/Beta catalyst; (d) TEM images with the corresponding element mapping of 5%Zr/Beta catalyst after regeneration.

### 3.4 Online Mass-Spectrometry Modulation Experiments

To further elucidate the reaction pathways for the conversion of the AE mixture to propene, online modulation experiments over 5%Zr/Beta catalyst were performed at 623 K, as depicted in Figure 8. For acetone-only conversion experiments (Figure 8a), an initial distinct trend in C4 olefin formation is observed, followed by an increase in crotonaldehyde and acetic acid, which indicates that aldol condensation occurs on the Zr/Beta catalyst. Notably, isopropanol exhibits no significant change upon the introduction of acetone, suggesting that the increase in propene is not attributable to isopropanol. Instead, this phenomenon may result from the partial cracking of isobutylene.^36,37^ In the case of pure ethanol conversion (Figure 8b), trends analogous to those of the acetone-only conversion experiments are detected, yielding ethylene as the dominant product with minor amounts of propene and butylene. This aligns with the catalytic results shown in Figure 4, indicating that ethanol dehydration is accompanied by secondary reactions leading to generation of higher olefins. When co-feeding an ethanol-acetone mixture (Figure 8c), a pronounced change in the isopropanol evolution is observed, exhibiting a volcano trend that coincides with rising propene production. This demonstrates that ethanol and acetone undergo the MPV reduction over the Zr/Beta catalyst to form isopropanol, followed by rapidly converting to propene. The formation of C4 olefins and ethylene can be attributed to the reactions analogous to those observed in the acetone-only and ethanol-only experiments.

**Figure 8:**
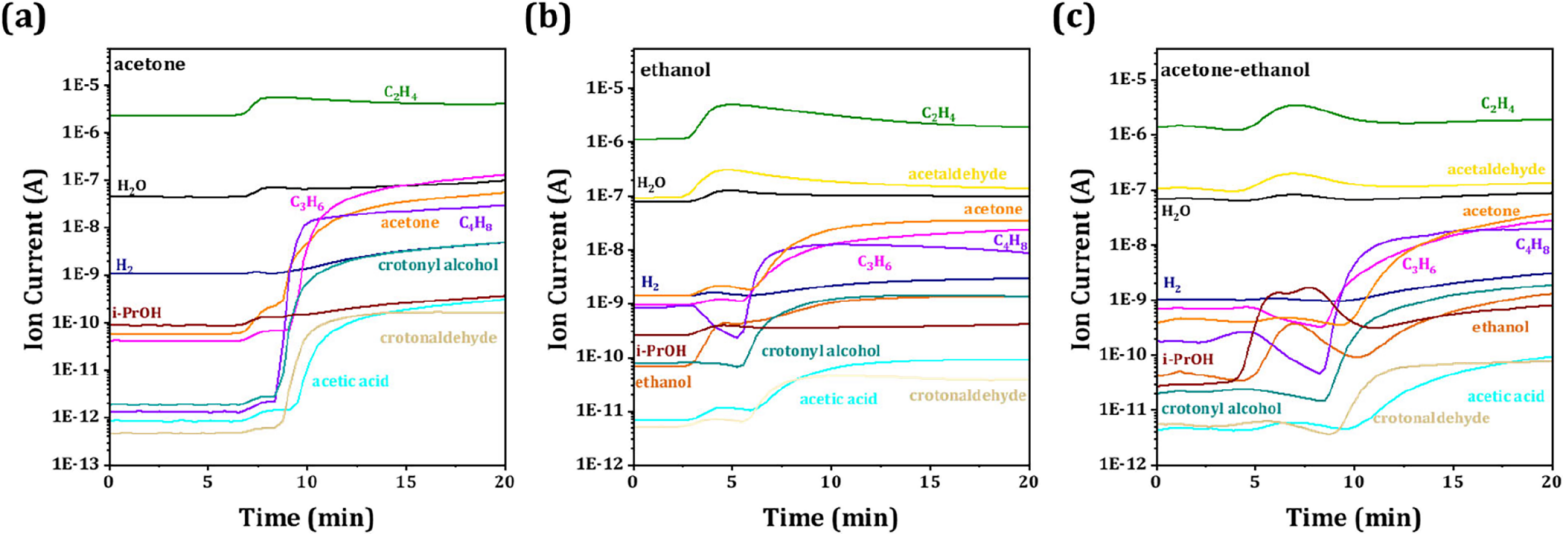
Online mass-spectrometry experiments with acetone (a), ethanol (b), and an acetone-ethanol mixture (c) over 5%Zr/Beta. Reaction conditions: temperature = 623 K, WHSV = 1.0 h^–1^, and TOS = 20 min.

Based on the catalytic results with spectroscopic characterization, a comprehensive reaction network and deactivation mechanism in the AE mixture conversion to propene over Zr/Beta catalysts have been proposed, as illustrated in Figure 9. The reaction proceeds via the MPV reduction of acetone with ethanol at Lewis acid sites (LASs) to generate an isopropanol intermediate, followed by dehydration to propene. Additionally, acetone undergoes a series of sequential reactions, including aldol condensation, dehydration, and cracking, to produce isobutene. The formation of isobutene is accompanied by the aldol condensation of acetone with intermediate dimethoxy species, yielding phorone-A and phorone-B.^30,38^ These unsaturated aldehydes, ketones, and aromatic compounds strongly adsorb onto the active sites, gradually leading to catalyst deactivation. Concurrently, ethanol undergoes dehydration to produce ethylene, which further contributes to the complexity of the reaction network.

**Figure 9:**
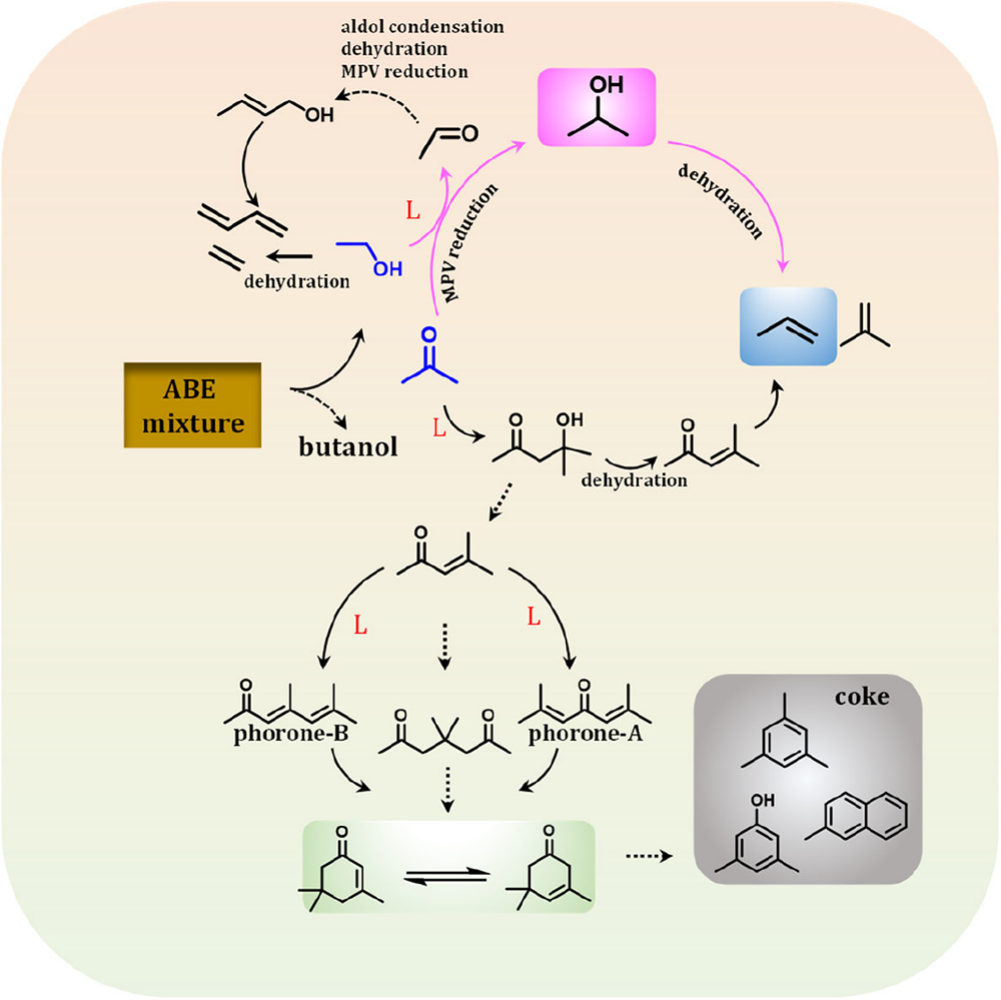
Proposed reaction pathway and the possible deactivation mechanism of the acetone-ethanol mixture to propene over the Zr/Beta catalyst.

## 4 Conclusion

Herein, a high-efficiency Zr/Beta zeolite catalyst was synthesized and employed in the conversion of an acetone-ethanol (AE) mixture into propene, bypassing energy-intensive extraction processes required in conventional biomass-derived 1-butanol upgrading systems. The AE mixture proceeds via the MPV reduction at Zr^4+^ Lewis acid sites to generate isopropanol, followed by a dehydration process at weakly acid sites (Si–OH) to produce propene over the Zr/Beta catalyst, which achieves a propene selectivity of up to 67% with a propene yield of 37.8%. Remarkably, the catalyst exhibits excellent regenerability, as coke deposits are readily removed through facile calcination in air, resulting in full recovery of catalytic performance. Online mass spectrometry was employed to elucidate the reaction pathway and deactivation mechanism, revealing key intermediates and coke formation dynamics. The process operates at mild reaction temperatures, effectively suppressing undesirable side reactions such as acetone self-condensation and CO_2_ emissions. Furthermore, unreacted substrates can be recycled, enhancing both industrial feasibility and carbon efficiency.
